# Computed Tomography Angiography-Derived Scores for Prediction of Chronic Total Occlusion Percutaneous Coronary Intervention Using the Hybrid Algorithm

**DOI:** 10.3390/jcdd11010003

**Published:** 2023-12-22

**Authors:** Antoni Zyśk, Rafał Wolny, Mariusz Kruk, Jacek Kwieciński, Artur Dębski, Umberto Barbero, Cezary Kępka, Marcin Demkow, Adam Witkowski, Maksymilian P. Opolski

**Affiliations:** 1 Department of Interventional Cardiology and Angiology, National Institute of Cardiology, 04-682 Warsaw, Polandmopolski@ikard.pl (M.P.O.); 2 Department of Coronary and Structural Heart Diseases, National Institute of Cardiology, 04-682 Warsaw, Poland; 3 Department of Cardiology, Santissima Annunziata Hospital, 12038 Savigliano, Italy

**Keywords:** coronary chronic total occlusion, coronary computed tomography angiography, percutaneous coronary intervention, hybrid algorithm

## Abstract

Whereas coronary computed tomography angiography (CCTA) exceeds invasive angiography for predicting the procedural outcome of chronic total occlusion (CTO) percutaneous coronary intervention (PCI), CCTA-derived scores have never been validated in the hybrid CTO PCI population. In this single-center, retrospective, observational study, we included 108 consecutive patients with 110 CTO lesions and preprocedural CCTA who underwent hybrid CTO PCI to assess the diagnostic accuracy of CCTA-derived scoring systems. Successful guidewire crossing within 30 min was set as the primary endpoint. The secondary endpoints were final procedural success and the need for using any non-antegrade wiring (AW) strategy within the hybrid algorithm. Time-efficient guidewire crossing and final procedural success were achieved in 53.6% and 89.1% of lesions, respectively, while in 36.4% of the procedures, any non-AW strategy was applied. The median J-CTO score was 1 (interquartile range (IQR): 0, 2), while the CT-RECTOR, KCCT, J-CTO_CCTA_, and RECHARGE_CCTA_ scores were 2 (IQR: 1, 3), 3 (IQR: 2, 5), 1 (IQR: 0, 3), and 2 (IQR: 1, 3), respectively. All scores were significantly higher in the lesions with failed versus successful time-efficient guidewire crossing. Although all of the CCTA-derived scores had numerically higher predictive values than the angiographic J-CTO score, no significant differences were noted between the scores in any of the analyzed study endpoints. High sensitivity of the CT-RECTOR and RECHARGE_CCTA_ scores (both 89.8%) for predicting successful guidewire crossing within 30 min, and high sensitivity (90.8%) of the KCCT score for predicting final procedural success, were noted. CCTA-derived scoring systems are accurate, noninvasive tools for the prediction of the procedural outcome of hybrid CTO PCI, and may aid in identifying the need for use of the hybrid algorithm.

## 1. Introduction

Percutaneous coronary intervention (PCI) in chronic total occlusions (CTOs) improves anginal symptoms [1] and myocardial perfusion [2] and might have favorable effects on prognosis [3]. In recent years, the introduction of newer procedural strategies (the retrograde approach and dissection and re-entry techniques) [4] as well as specialized devices [5,6] has resulted in substantial improvements in CTO recanalization rates (~90% in some luminary centers). Specifically, the application of a systematic algorithm comprising multiple techniques and devices (called the “hybrid approach”) is widely employed to cross the CTO lesion in a time-efficient and safe manner [7]. Nevertheless, the incidence of attempted CTO PCI is still relatively low, with wide variability between centers. Specifically, the uncertainty of procedural success and the time-consuming nature of CTO PCI remain a strong barrier to its wider adoption and accessibility [8]. To quantify the technical difficulty of the CTO lesion and alleviate the uncertainty before the procedure, well-established predictive scores derived from coronary angiography [8,9] and coronary computed tomography angiography (CCTA) [10,11,12,13] have been developed and widely adopted in clinical practice. Although CCTA is increasingly employed in the diagnostic workup of patients with coronary artery disease, and has superior diagnostic accuracy over invasive angiography for the prediction of the CTO difficulty level prior to PCI [12,14], it has never been validated in patients undergoing CTO PCI in accord with the hybrid algorithm. We thus aimed to provide a comprehensive diagnostic accuracy analysis of CCTA-derived scores for the prediction of hybrid CTO PCI.

## 2. Materials and Methods

### 2.1. Study Design and Population

In this single-center, retrospective, observational study, we enrolled 108 consecutive patients with at least one CTO lesion in the native coronary artery who underwent preprocedural CCTA within 1 year prior to elective CTO PCI performed between October 2018 and December 2022. The study protocol was approved by the institutional ethics committee, and informed consent was waived. CTO was defined as a TIMI (thrombolysis in myocardial infarction) flow grade 0 within the native coronary artery estimated to be of at least 3 months’ duration based on either previous angiography or clinical history [15]. The exclusion criterion was an in-stent CTO. Clinical and demographic characteristics (including reattempt of previously failed CTO PCI, duration of CTO, and a history of coronary artery bypass grafting necessary to calculate the predictive scores) were obtained from the electronic medical records. 

### 2.2. Study Endpoints

The primary endpoint was time-efficient guidewire crossing through the CTO lesion, defined as successful crossing within 30 min after the first guidewire was inserted into the CTO vessel [8]. This is commonly perceived as the most objective parameter, corresponding to the level of difficulty intrinsic to the CTO lesion and minimizing the operator-related variability. The secondary endpoints were (1) final procedural success, defined as successful guidewire crossing through the CTO at any time, with restoration of flow (<50% residual stenosis and TIMI flow grade 3), and (2) the need for the use of any non-antegrade wiring (AW) strategy within the hybrid algorithm.

### 2.3. CCTA Protocol, Image Reconstruction, and Analysis

CCTA was performed with two computed tomographic scanners (Somatom Definition Flash and Somatom Force, Siemens, Erlangen, Germany). Unless contraindicated, sublingual nitroglycerin (0.8 mg) was administered and, in case of heart rate > 65 beats/min, intravenous metoprolol (sequence of 5 mg) was given prior to the CT scan. A 60 to 120 mL bolus of iodinated contrast material (Iomeron 400, Bracco Altana Pharma, Konstanz, Germany) was administered intravenously at a rate of 6 mL/s (Flash) or 4.5 mL/s (Force). A retrospectively electrocardiogram-gated or prospectively electrocardiogram-triggered protocol was used with a beam collimation of 128 × 0.6 mm (Flash) or 192 × 0.6 mm (Force) and a tube voltage of 70–120 kV, adjusted manually depending on the body mass index. Image data were reconstructed in mid-to-end diastole (65% to 75% of R-R interval) and systole (35% to 45% of R-R interval) with 0.6 mm slice thickness and 0.4 mm increment. All CCTA data were analyzed offline using a dedicated software tool (Syngo, Siemens Healthineers, Erlangen, Germany) by an experienced reader (A.Z.) with 5 years of experience in CCTA, blinded to the clinical and angiographic data. Interobserver variability was assessed in 15% of all the CTO lesions by a second experienced observer (M.P.O.) with 15 years of experience in CCTA. The CCTA datasets were evaluated using axial images, cross-sectional views, curved multiplanar reformation (curved MPR), and 3-dimensional maximum intensity projections. For all CTO lesions, the previously described CT-derived scores (CT-RECTOR, KCCT, J-CTO_CCTA_, and RECHARGE_CCTA_) were calculated [10,11,12,13]. The CT-RECTOR score assigns 1 point to each of the following variables: multiple occlusions, blunt cap shape at the entry or exit site of the occlusion, severe calcification, bending > 45°, duration > 12 months or unknown, and reattempt procedure. The KCCT score assigns 1 point to each of the following variables: proximal blunt cap shape, proximal adjacent side branch, occlusion length ≥ 15 mm, bending > 45°, duration > 12 months or unknown, reattempt procedure, and severe peripheral calcification; 2 points are assigned to central calcification. The J-CTO_CCTA_ assigns 1 point to each of the following variables: proximal blunt cap shape, occlusion length ≥ 20 mm, severe calcification, bending > 45°, and reattempt procedure. The RECHARGE_CCTA_ assigns 1 point to each of the following variables: proximal blunt cap shape, occlusion length ≥ 20 mm, severe calcification, bending > 45°, diseased distal landing zone, and previous bypass grafting to CTO vessel. Proximal cap shape was classified as either tapered or blunt. Total occlusion length was measured on curved MPR, as previously reported [16]. A lesion was classified as tortuous when at least one bend of > 45° within a CTO segment was present. Depending on the score, calcification was evaluated differently. Severe calcification was defined as the presence of calcified area ≥ 50% of the vessel’s cross-sectional area (CSA) within the CTO segment in the CT-RECTOR, J-CTO_CCTA_, and RECHARGE_CCTA_ scores. According to the KCCT score, calcification was described as either severe peripheral (maximal encircling ≥ 180° and calcified area ≥ 50% of CSA) or central (maximal encircling equal to 360° and calcified area equal to 100% of CSA) [11]. Diseased distal landing zone was defined as the presence of lumen stenosis > 50% distal to the occluded segment or a distal lumen diameter of <2 mm [13]. Multiple occlusion was defined as the presence of ≥2 complete interruptions of the contrast opacification separated by a contrast-enhanced segment of ≥5 mm [10].

### 2.4. Coronary Angiography and CTO PCI

Coronary angiography and CTO PCI were performed using a Siemens Artis zee fluoroscopy unit (Siemens Medical Solutions, Erlangen, Germany). All procedures were performed by a team of two highly experienced CTO operators (minimum of 70 CTO PCI annually) according to the hybrid algorithm. In this regard, antegrade wiring (AW), antegrade dissection and re-entry (ADR), retrograde wiring (RW), and retrograde dissection and reentry (RDR) strategies were applied. For all lesions, the angiography-based J-CTO score was calculated as previously described [8].

### 2.5. Statistical Analysis

Continuous variables are presented as the mean and standard deviation or median and interquartile range, as appropriate. Categorical variables are presented as absolute counts with percentage. Continuous variables were compared using the Student’s *t*-test or Mann–Whitney U test, as appropriate, and differences in categorical variables were measured with the Fisher exact test. The performance of the CTO predictive scores was evaluated by comparing the receiver operating characteristic (ROC) curves with a corresponding area under the curve (AUC) using the DeLong method [17]. The intraobserver and interobserver agreement was analyzed using the kappa statistics. Statistical significance was set at a *p*-value of <0.05. All statistical analyses were performed using Python 3 packages (NymPy, Pandas, SciPy, sci-kit learn) and MedCalc (version 20.2, MedCalc Software, Mariakerke, Belgium).

## 3. Results

### 3.1. Clinical Characteristics

**Figure 1:**
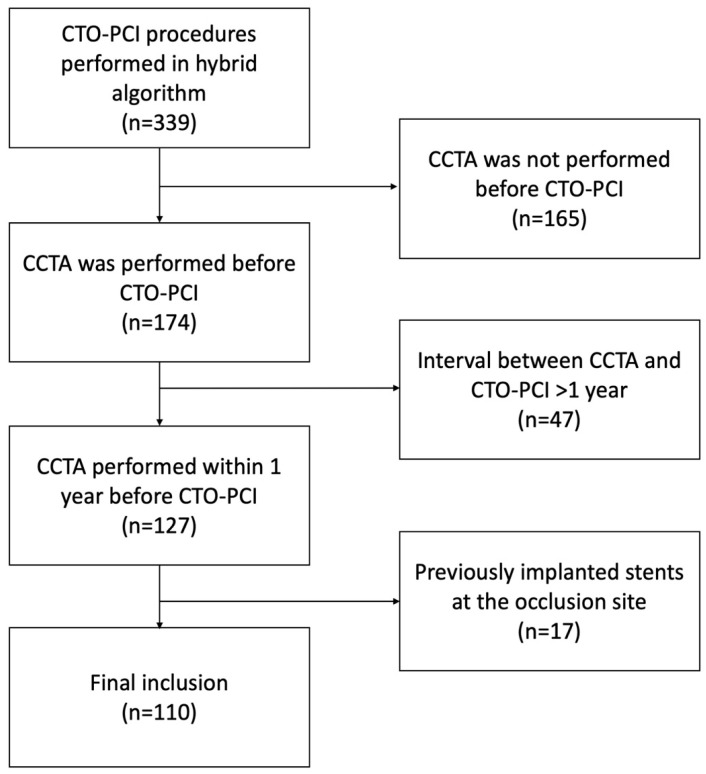
Patient flow chart.

From the total of 339 CTO PCI performed between October 2018 and December 2022, 212 CTO lesions were excluded due to a lack of preprocedural CCTA within 1 year prior to the CTO recanalization attempt, and 17 CTO lesions were excluded due to previously implanted stents at the occlusion site. Finally, 108 patients (median age of 67, 80% male) with 110 CTO lesions were enrolled (Figure 1). CTO PCI was performed at a median interval of 24 days (IQR: 3 to 93 days) after CCTA. There were no significant differences in clinical characteristics between the successful and failed time-efficient guidewire crossing groups (Table 1).

### 3.2. Angiographic and Procedural Characteristics

The median J-CTO score was 1 (IQR: 0, 2), with a successful guidewire crossing at any time in 90.9% lesions, and a median time of successful guidewire crossing of 20 min (IQR: 8 to 83.2 min) (Supplementary Figure S1). Time-efficient guidewire crossing and final procedural success (Supplementary Figures S2 and S3) were achieved in 53.6% and 89.1% of lesions, respectively, whereas in 36.4% of the procedures, any non-AW strategy was applied. Antegrade wiring, ADR, RW, and RDR strategies were applied in 96.4%, 23.6%, 29.1%, and 8.2% of all procedures, respectively. If AW was the initial but eventually failed strategy, the median time of changing the strategy from AW to any non-AW strategy was 27 min (IQR: 19 to 52 min). Overall, AW, ADR, RW, and RDR were the final successful strategies in 64.6%, 7.3%, 10.9%, and 8.2% of all procedures, respectively. According to coronary angiography, time-efficient guidewire crossing was achieved less frequently in longer occlusions as well as in CTO lesions with higher tortuosity, calcification, and blunt proximal cap. Overall, the J-CTO score was significantly higher in lesions with failed versus successful time-efficient guidewire crossings (2 vs. 1, *p* < 0.001). Angiographic and procedural characteristics are shown in Table 2.

### 3.3. Computed Tomographic Characteristics

**Figure 2:**
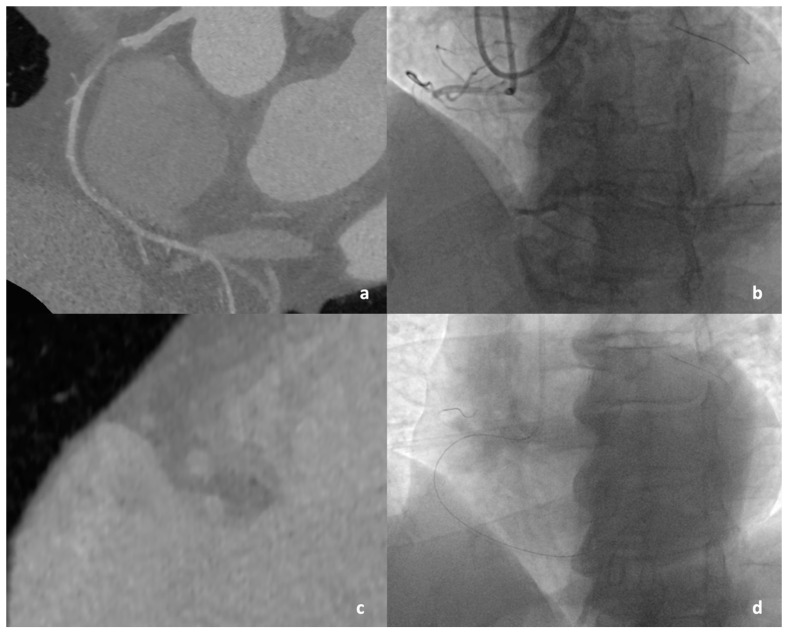
Successful time-efficient guidewire crossing. (**a**) Short, noncalcified lesion presented on MPR. (**b**) Angiogram of CTO lesion before insertion of first guidewire into the vessel. (**c**) Cross-section projection of CTO lesion without calcification. (**d**) Successful antegrade guidewire crossing within 30 min.

**Figure 3:**
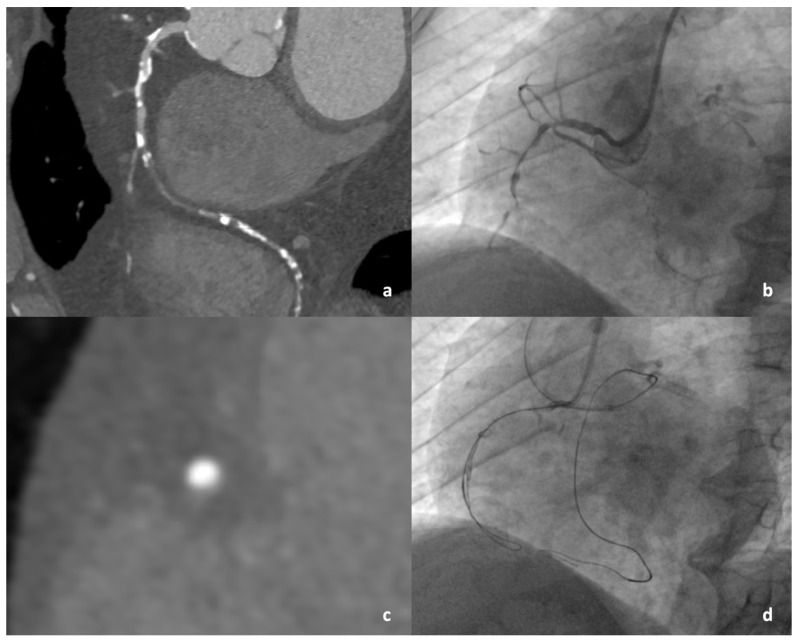
Failed time-efficient guidewire crossing. (**a**) Long and calcified lesion presented on MPR. (**b**) Angiogram of CTO lesion before insertion of first guidewire into the vessel. (**c**) Cross-section projection of CTO lesion with severe calcification. (**d**) No successful guidewire crossing within 30 min. Antegrade wire in subintimal space and attempted guidewire crossing with retrograde approach through septal collaterals.

The time-efficient guidewire-failure group showed longer occlusions with higher tortuosity and higher prevalence of blunt proximal cap, proximal adjacent side branch, multiple occlusions, as well as severe calcification on CCTA (Figure 2 and Figure 3). The median values of the CT-RECTOR, KCCT, J-CTO_CCTA_, and RECHARGE_CCTA_ scores were 2 (IQR: 1, 3), 3 (IQR: 2, 5), 1 (IQR: 0, 3), and 2 (IQR: 1, 3), respectively. All CCTA-derived scores were significantly higher in the failed versus the successful time-efficient guidewire crossing group. Computed tomographic characteristics are shown in Table 3. Intraobserver and interobserver variability for categorical parameters assessed using both CCTA and invasive coronary angiography revealed substantial to excellent agreement with mean Cohen’s kappa values of 0.76 (IQR: 0.65–0.88) and 0.73 (IQR: 0.65–0.76), respectively. 

### 3.4. Diagnostic Accuracy of CCTA-Derived Scores

**Figure 4:**
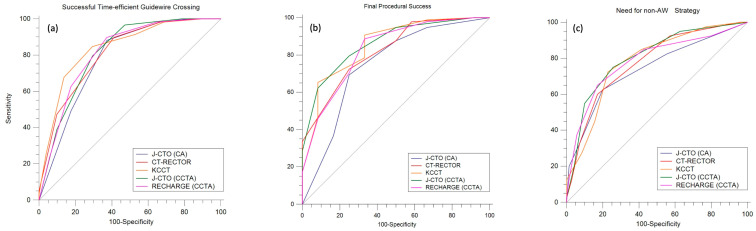
Comparison of the ROC curves of scoring systems, predicting (**a**) successful time-efficient guidewire crossing, (**b**) final procedural success, and (**c**) the need for non-AW strategy.

The ROC curves with corresponding AUCs of the evaluated scores are shown in Figure 4 and Table 4. For predicting time-efficient guidewire crossing, the KCCT score had the numerically highest AUC of 0.839 (95% CI: 0.757 to 0.902) with the best cut-off value of ≤3 points. Both the CT-RECTOR score and the RECHARGE_CCTA_ score had the highest sensitivity (89.8%), while the KCCT score and the J-CTO_CCTA_ score had the highest specificity (70.6%) for the prediction of time-efficient guidewire crossing. For predicting the final procedural success, the J-CTO_CCTA_ score had the numerically highest AUC of 0.864 (95% CI: 0.785 to 0.922) with the best cut-off value of ≤2 points. The KCCT score had the highest sensitivity (90.8%), while the CT-RECTOR, J-CTO_CA_, and J-CTO_CCTA_ scores had the highest specificity (75%) for the prediction of final procedural success. For predicting the need for any non-AW strategy, the J-CTO_CCTA_ score had the numerically highest AUC of 0.806 (95% CI: 0.72 to 0.875) with the best cut-off value of >1 point and the highest sensitivity (72.5%), while the CT-RECTOR score and the RECHARGE_CCTA_ score had the highest specificity (82.9%).

Although all of the CCTA-derived scores had numerically higher predictive values than the angiographic J-CTO scores, considering each of the study endpoints, no significant differences were noted between the scores. Similarly, there were no significant differences between the CCTA-derived scores for the prediction of any of the study endpoints.

## 4. Discussion

To our knowledge, this is the first comprehensive study to evaluate the diagnostic accuracy of CCTA-derived scoring systems for the prediction of CTO PCI performed in the hybrid algorithm approach. Considering all study endpoints (including time-efficient guidewire crossing, final procedural success, and the need for any non-AW strategy), the CCTA-derived scores had numerically higher diagnostic accuracies compared with the angiography-based J-CTO score; however, none of the differences between the scores were statistically significant. In addition, while we showed particularly high sensitivity of the CT-RECTOR and the RECHARGE_CCTA_ scores (both 89.8%) for the prediction of time-efficient guidewire crossing, the KCCT score had the highest sensitivity (90.8%) for predicting final procedural success. Notably, our findings may be considered fairly reproducible to other hybrid CTO PCI populations, given the relatively high prevalence of the antegrade dissection and reentry and the retrograde approach strategies (24% and 31%, respectively) in our study. 

Time-efficient (within 30 min) guidewire crossing is a well-established and objective parameter for the assessment of CTO difficulty level, reflecting both the procedural duration as well as the resources use [8]. Considering this endpoint, the KCCT score had the numerically highest AUC (cut-off value of ≤3 points) and the highest specificity (70.6%), whereas both the CT-RECTOR score and the RECHARGE_CCTA_ score (cut-off value of ≤2 points) showed the highest sensitivity (89.8%). Thus, and similar to prior reports on non-hybrid CTO PCI [10], we corroborate the application of CCTA-derived scores with high sensitivity to accurately predict longer procedures irrespective of the use of the hybrid algorithm.

For prediction of the final procedural success, the J-CTO_CCTA_ score had the numerically highest AUC with the best cut-off value of ≤2 points. Noteworthy is the high sensitivity (90.8%) of the KCCT score for the cut-off point ≤5, indicating accurate predictability of failed procedural success in lesions with high complexity scores referred for CTO PCI, according to the hybrid algorithm. Given that CTO PCI represents one of the most challenging procedures in interventional cardiology, with relatively lower success rates and higher complications than non-CTO PCI, preprocedural evaluation of the chances for final procedural success may alleviate operator uncertainty and improve the benefit-to-risk assessment for judicious patient selection for CTO PCI. 

Whereas CTO scoring systems were originally developed to predict time-efficient guidewire crossing as well as final procedural success, we propose a novel endpoint, defined as the need for any non-AW strategy corresponding to the use of the hybrid algorithm. Of particular interest, both the CT-RECTOR score and the RECHARGE_CCTA_ score showed the highest specificity (both 82.9%), both with cut-off points of > 2. We believe that the satisfactory specificity of the above-mentioned scores might aid in gauging the need for the use of the hybrid algorithm and consequently improve patients’ referrals to highly experienced CTO operators acquainted with all the hybrid strategies. On the contrary, relatively easier CTO lesions may be used for training purposes for less-experienced operators specialized in AW only. 

Prior studies reported that detailed noninvasive assessment and quantification of calcification within the occlusion site based on CCTA may improve diagnostic accuracy for predicting CTO PCI procedural success [18,19]. Beyond preprocedural planning, CCTA can provide periprocedural guidance during CTO PCI in the catheterization laboratory by means of CT co-registration [20]. This approach has been found particularly useful for resolving proximal cap ambiguity, disclosing CTO vessel course and calcium deposits, as well as providing guidance on the most optimal reentry site during ADR. Of note, a prior randomized study demonstrated that preprocedural CCTA might increase the CTO PCI success rate in lesions with higher difficulty scores than angiography guidance alone [21]. Moreover, CT was verified not only as an accurate anatomical imaging modality for the detailed morphological assessment of CTO [22] but also for myocardial perfusion, lending support for one-stop-shop examination in the context of CTO patients considered for revascularization therapies [23,24]. In this regard, and along with current guidelines [25], our results substantiate the clinical application of CCTA prior to CTO PCI.

## 5. Conclusions

CCTA-derived scoring systems are accurate, noninvasive tools for the prediction of time-efficient guidewire crossing, final procedural success, and the need for any non-AW strategy in CTO patients undergoing PCI within the hybrid algorithm. We thus advocate calculating CCTA-derived scoring systems in patients with preprocedural CCTA data availability who are being considered for hybrid CTO PCI.

## 6. Limitations

Our study had some limitations. First, this was a retrospective, single-center, and observational report with the potential for selection bias. Second, by virtue of the limited number of preprocedural CCTA scans, we included a relatively small study population. Thus, further analyses in larger multi-center studies are required to confirm our findings. Finally, the interval between preprocedural CCTA and CTO PCI was extended up to 1 year prior to CTO PCI, and 63% of patients who underwent hybrid CTO PCI in our center were excluded due to lack of CCTA scan within this period.

## Figures and Tables

**Table 1:** Clinical characteristics.

	Total (*n* = 110)	Time-Efficient GW Crossing (*n* = 59)	No Time-Efficient GW Crossing (*n* = 51)	*p*-Value
Age	67.0 (60.3–72.0)	67.0 (56.9–72.0)	67.0 (62.0–69.6)	0.196
Male	88 (80.0%)	46 (78.0%)	42 (82.4%)	0.637
BMI	28.82 (26.76, 31.59)	28.93 (26.44, 31.82)	28.73 (27.31, 31.16)	0.767
Family history of CAD	13 (11.8%)	8 (13.6%)	5 (9.8%)	0.572
Current smoker	27 (24.5%)	15 (25.4%)	12 (23.5%)	1.000
Hypertension	91 (82.7%)	51 (86.4%)	40 (78.4%)	0.317
Dyslipidemia	92 (83.6%)	52 (88.1%)	40 (78.4%)	0.202
Diabetes mellitus	34 (30.9%)	15 (25.4%)	19 (37.3%)	0.217
Renal disease	16 (14.5%)	8 (13.6%)	8 (15.7%)	0.792
Creatinine	1.0 (0.9–1.1)	0.9 (0.9–1.0)	1.0 (0.8–1.1)	0.367
eGFR	89.8 (71.9–114.5)	93.6 (77.2–112.6)	88.0 (69.1–114.9)	0.642
PAD	29 (26.4%)	13 (22.0%)	16 (31.4%)	0.286
TIA/stroke	9 (8.2%)	6 (10.2%)	3 (5.9%)	0.500
Heart failure	24 (21.8%)	10 (16.9%)	14 (27.5%)	0.248
LVEF ≤ 40	16 (14.5%)	9 (15.3%)	7 (13.7%)	1.000
COPD	9 (8.2%)	5 (8.5%)	4 (7.8%)	1.000
CCS	2.0 (0.0–3.0)	2.0 (0.5–3.0)	2.0 (0.0–3.0)	0.862
NYHA	0.0 (0.0–2.0)	0.0 (0.0–2.0)	0.0 (0.0–2.0)	0.213
NYHA ≥ II	34 (30.9%)	16 (27.1%)	18 (35.3%)	0.411
CCS ≥ 2	74 (67.3%)	40 (67.8%)	34 (66.7%)	1.000
Prior MI	53 (48.2%)	25 (42.4%)	28 (54.9%)	0.251
Prior CABG	22 (20.0%)	8 (13.6%)	14 (27.5%)	0.094
Prior PCI	66 (60.0%)	35 (59.3%)	31 (60.8%)	1.000
Unknown or >12 months’ duration of CTO	98 (89.1%)	50 (84.7%)	48 (94.1%)	0.137
Reattempt at CTO	24 (21.8%)	11 (18.6%)	13 (25.5%)	0.489
Grafted CTO vessel	19 (17.3%)	8 (13.6%)	11 (21.6%)	0.317

GW—guidewire, BMI—body mass index, CAD—coronary artery disease, eGFR—estimated glomerular filtration rate, PAD—peripheral artery disease, TIA—transient ischemic attack, LVEF—left ventricular ejection fraction, COPD—chronic obstructive pulmonary disease, CCS—Canadian Cardiovascular Society, NYHA—New York Heart Association, MI—myocardial infarction, CABG—coronary artery bypass graft, PCI—percutaneous coronary intervention, CTO—chronic total occlusion.

**Table 2:** Angiographic and procedural characteristics.

	Total (*n* = 110)	Time-Efficient GW Crossing (*n* = 59)	No Time-Efficient GW Crossing (*n* = 51)	*p*-Value
CTO in RCA	53 (48.2%)	26 (44.1%)	27 (52.9%)	0.444
CTO in LAD	44 (40.0%)	24 (40.7%)	20 (39.2%)	1.000
CTO in CX	13 (11.8%)	9 (15.3%)	4 (7.8%)	0.255
Successful GW crossing at any time	100 (90.9%)	59 (100.0%)	41 (80.4%)	<0.001
Restoration of TIMI 3 flow	98 (89.1%)	59 (100.0%)	39 (76.5%)	<0.001
Any AW	106 (96.4%)	59 (100.0%)	47 (92.2%)	0.043
Any ADR	26 (23.6%)	1 (1.7%)	25 (49.0%)	<0.001
Any RW	32 (29.1%)	1 (1.7%)	31 (60.8%)	<0.001
Any RDR	9 (8.18%)	0 (0.00%)	9 (17.65%)	0.001
Any DART	29 (26.4%)	1 (1.7%)	28 (54.9%)	<0.001
Retrograde approach	34 (30.9%)	1 (1.7%)	33 (64.7%)	<0.001
Any non-AW strategy	40 (36.4%)	2 (3.4%)	38 (74.5%)	<0.001
No. strategies applied	1.0 (1.0–2.0)	1.0 (1.0–1.0)	2.0 (1.0–3.0)	<0.001
Successful AW	71 (64.5%)	57 (96.6%)	14 (27.5%)	<0.001
Successful ADR	8 (7.3%)	1 (1.7%)	7 (13.7%)	0.024
Successful RW	12 (10.9%)	1 (1.7%)	11 (21.6%)	0.001
Successful RDR	9 (8.2%)	0 (0.0%)	9 (17.6%)	0.001
Change from AW to any non-AW strategy (min)	27.0 (19.0–52.0)	14.5 (10.2–18.8)	30.0 (19.0–53.0)	0.248
Time of successful GW crossing (min)	20.0 (8.0–83.2)	9.0 (5.5–15.5)	110.0 (67.0–142.0)	<0.001
Duration of procedure (min)	142.5 (92.2–209.5)	97.0 (76.5–137.0)	211.0 (162.5–233.5)	<0.001
Fluoroscopy time (min)	43.5 (27.4–73.4)	29.0 (19.4–42.2)	74.0 (55.8–94.7)	<0.001
Radiation dose (mGy)	1506.5 (794.0–2594.5)	917.0 (501.9–1624.5)	2531.0 (1436.0–3254.0)	<0.001
Contrast volume (mL)	200.0 (132.5–200.0)	150.0 (100.0–200.0)	200.0 (200.0–250.0)	<0.001
Blunt proximal cap	39 (35.5%)	10 (16.9%)	29 (56.9%)	<0.001
Calcification	17 (15.5%)	4 (6.8%)	13 (25.5%)	0.008
Tortuosity (°)	25.0 (15.0–38.3)	22.4 (14.1–32.5)	30.0 (15.8–53.5)	0.005
Tortuosity > 45°	23 (20.9%)	6 (10.2%)	17 (33.3%)	0.004
Occlusion length (mm)	11.3 (7.0–22.7)	9.0 (6.4–12.9)	21.0 (11.2–27.3)	<0.001
Length ≥ 20 mm	34 (30.9%)	7 (11.9%)	27 (52.9%)	<0.001
J-CTO_CA_ Score	1.0 (0.0–2.0)	1.0 (0.0–1.0)	2.0 (1.0–3.0)	<0.001

GW—guidewire, CTO—chronic total occlusion, RCA—right coronary artery, LAD—left anterior descending artery, CX—circumflex artery, TIMI—thrombolysis in myocardial infarction, AW—antegrade wiring, ADR—antegrade dissection and reentry, RW—retrograde wiring, RDR—retrograde dissection and reentry, DART—dissection and reentry technique, CA—coronary angiography.

**Table 3:** Computed tomographic characteristics.

	Total (*n* = 110)	Time-Efficient GW Crossing (*n* = 59)	No Time-Efficient GW Crossing (*n* = 51)	*p*-Value
Blunt cap	40 (36.4%)	11 (18.6%)	29 (56.9%)	<0.001
Proximal adjacent SB	57 (51.8%)	25 (42.4%)	32 (62.7%)	0.037
Occlusion length	15.6 (9.3–23.9)	12.0 (8.6–17.0)	22.6 (16.4–28.9)	<0.001
Occlusion length ≥ 15 mm	56 (50.9%)	16 (27.1%)	40 (78.4%)	<0.001
Occlusion length ≥ 20 mm	39 (35.5%)	9 (15.3%)	30 (58.8%)	<0.001
Tortuosity (°)	27.0 (17.2–40.0)	24.0 (16.5–34.5)	32.0 (19.5–52.0)	0.001
Tortuosity > 45°	23 (20.9%)	3 (5.1%)	20 (39.2%)	<0.001
Any calcification	89 (80.9%)	46 (78.0%)	43 (84.3%)	0.470
Calcium in entry of CTO	56 (50.9%)	30 (50.8%)	26 (51.0%)	1.000
Calcium in body of CTO	59 (53.6%)	26 (44.1%)	33 (64.7%)	0.036
Calcium in exit of CTO	53 (48.2%)	24 (40.7%)	29 (56.9%)	0.126
Calcium ≥ 50% CSA	45 (40.9%)	16 (27.1%)	29 (56.9%)	0.002
Calcium 100% CSA	18 (16.4%)	3 (5.1%)	15 (29.4%)	0.001
Multiple occlusions	16 (14.5%)	3 (5.1%)	13 (25.5%)	0.003
Diseased distal landing zone	55 (50.0%)	27 (45.8%)	28 (54.9%)	0.445
Proximal reference lumen area	6.6 (4.8–8.9)	6.1 (4.7–8.6)	7.3 (5.5–9.5)	0.224
Proximal reference maximal lumen diameter	3.1 (2.7–3.7)	3.0 (2.6–3.4)	3.2 (2.8–3.7)	0.088
Proximal reference minimal lumen diameter	2.7 (2.3–3.2)	2.6 (2.2–3.1)	2.8 (2.5–3.2)	0.124
Distal reference lumen area	4.5 (3.3–5.9)	4.5 (3.4–5.8)	4.0 (3.2–6.0)	0.952
Distal reference maximal lumen diameter	2.5 (2.1–3.0)	2.7 (2.1–3.0)	2.5 (2.1–3.0)	0.754
Distal reference minimal lumen diameter	2.1 (1.9–2.6)	2.1 (1.9–2.6)	2.1 (1.8–2.5)	0.673
Maximal vessel area within occlusion site	13.0 (8.8–18.0)	13.0 (8.7–18.0)	12.5 (9.3–18.0)	0.408
Remodeling index	1.3 (1.0–1.7)	1.3 (1.0–1.6)	1.3 (0.9–1.8)	0.962
CCTA-derived CTO scores				
CT-RECTOR score	2.0 (1.0–3.0)	2.0 (1.0–2.0)	3.0 (2.0–4.0)	<0.001
KCCT score	3.0 (2.0–5.0)	2.0 (1.5–3.0)	4.0 (3.0–6.0)	<0.001
J-CTO_CCTA_ score	1.0 (0.0–3.0)	1.0 (0.0–1.0)	3.0 (1.0–3.0)	<0.001
RECHARGE_CCTA_ score	2.0 (1.0–3.0)	1.0 (1.0–2.0)	3.0 (2.0–4.0)	<0.001

GW—guidewire, SB—side branch, CTO—chronic total occlusion, CSA—cross-sectional area, CCTA—coronary computed tomography angiography.

**Table 4:** Comparison of AUC and the best cut-off values between the CCTA-derived scores and the angiographic J-CTO score.

	AUC	AUC CI 95%	Criterion (Youden Index)	Sensitivity	Specificity	*p* (Area = 0.5)
Successful time-efficient guidewire crossing						
J-CTO_CA_	0.784	0.696–0.857	≤1	88.14	62.75	<0.001
CT-RECTOR	0.813	0.727–0.881	≤2	89.83	58.82	<0.001
KCCT	0.839	0.757–0.902	≤3	84.75	70.59	<0.001
J-CTO_CCTA_	0.815	0.73–0.883	≤1	79.66	70.59	<0.001
RECHARGE_CCTA_	0.817	0.731–0.884	≤2	89.83	62.75	<0.001
Final procedural success						
J-CTO_CA_	0.748	0.657–0.826	≤1	69.39	75	0.004
CT-RECTOR	0.824	0.739–0.89	≤2	72.45	75	<0.001
KCCT	0.855	0.775–0.915	≤5	90.82	66.67	<0.001
J-CTO_CCTA_	0.864	0.785–0.922	≤2	79.59	75	<0.001
RECHARGE_CCTA_	0.832	0.748–0.896	≤3	88.78	66.67	<0.001
Need for any non-AW strategy						
J-CTO_CA_	0.741	0.648–0.819	>1	62.5	80	<0.001
CT-RECTOR	0.777	0.688–0.851	>2	60	82.86	<0.001
KCCT	0.783	0.695–0.856	>3	72.5	77.14	<0.001
J-CTO_CCTA_	0.806	0.72–0.875	>1	75	74.29	<0.001
RECHARGE_CCTA_	0.785	0.697–0.858	>2	65	82.86	<0.001

AUC—area under curve, CI—confidence interval, CA—coronary angiography, CCTA—coronary computed tomography angiography, AW—antegrade wiring.

## Data Availability

Data are unavailable due to patient confidentiality.

## Supplementary Materials

The following supporting information can be downloaded at: https://www.mdpi.com/article/10.3390/jcdd11010003/s1, Figure S1: Time to successful CTO guidewire crossing depending on number of points in J-CTO_CA_, CT-RECTOR, KCCT, J-CTO_CCTA_, and RECHARGE_CCTA_ scoring systems.; Figure S2: Time-efficient guidewire crossing and final procedural success rates depending on number of points in J-CTO_CA_, CT-RECTOR, KCCT, J-CTO_CCTA_, and RECHARGE_CCTA_ scoring systems.; Figure S3: Time-efficient guidewire crossing and final procedural success rates depending on difficulty category based on J-CTO_CA_, CT-RECTOR, KCCT, J-CTO_CCTA_, and RECHARGE_CCTA_ scoring systems.
