# Supplementary material for: Computed Tomography Angiography-Derived Scores for Prediction of Chronic Total Occlusion Percutaneous Coronary Intervention Using the Hybrid Algorithm
Source: J Cardiovasc Dev Dis. 2023 Dec 22;11(1):3. doi: 10.3390/jcdd11010003 (PMC10817054; doi:10.3390/jcdd11010003)

Figure S1. Time to successful CTO guidewire crossing depending on number of points in (**a**) J-CTO$_{CA}$, (**b**) CT-RECTOR, (**c**) KCCT, (**d**) J-CTO$_{CCTA}$, and (**e**) RECHARGE$_{CCTA}$ scoring systems.

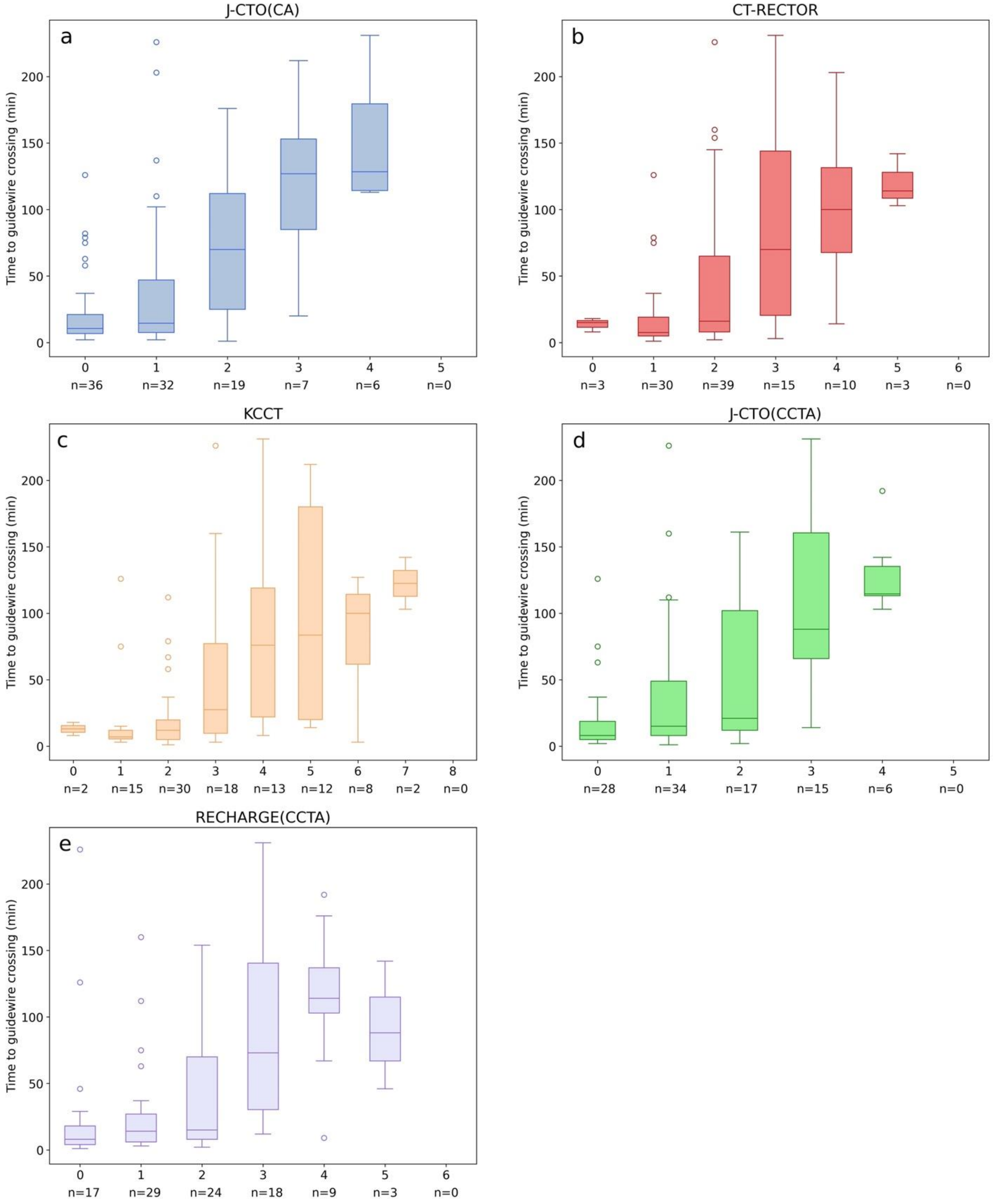

Figure S2. Time-efficient guidewire crossing and final procedural success rates depending on number of points in (**a**) J-CTO$_{CA}$, (**b**) CT-RECTOR, (**c**) KCCT, (**d**) J-CTO$_{CCTA}$, and (**e**) RECHARGE$_{CCTA}$ scoring systems.

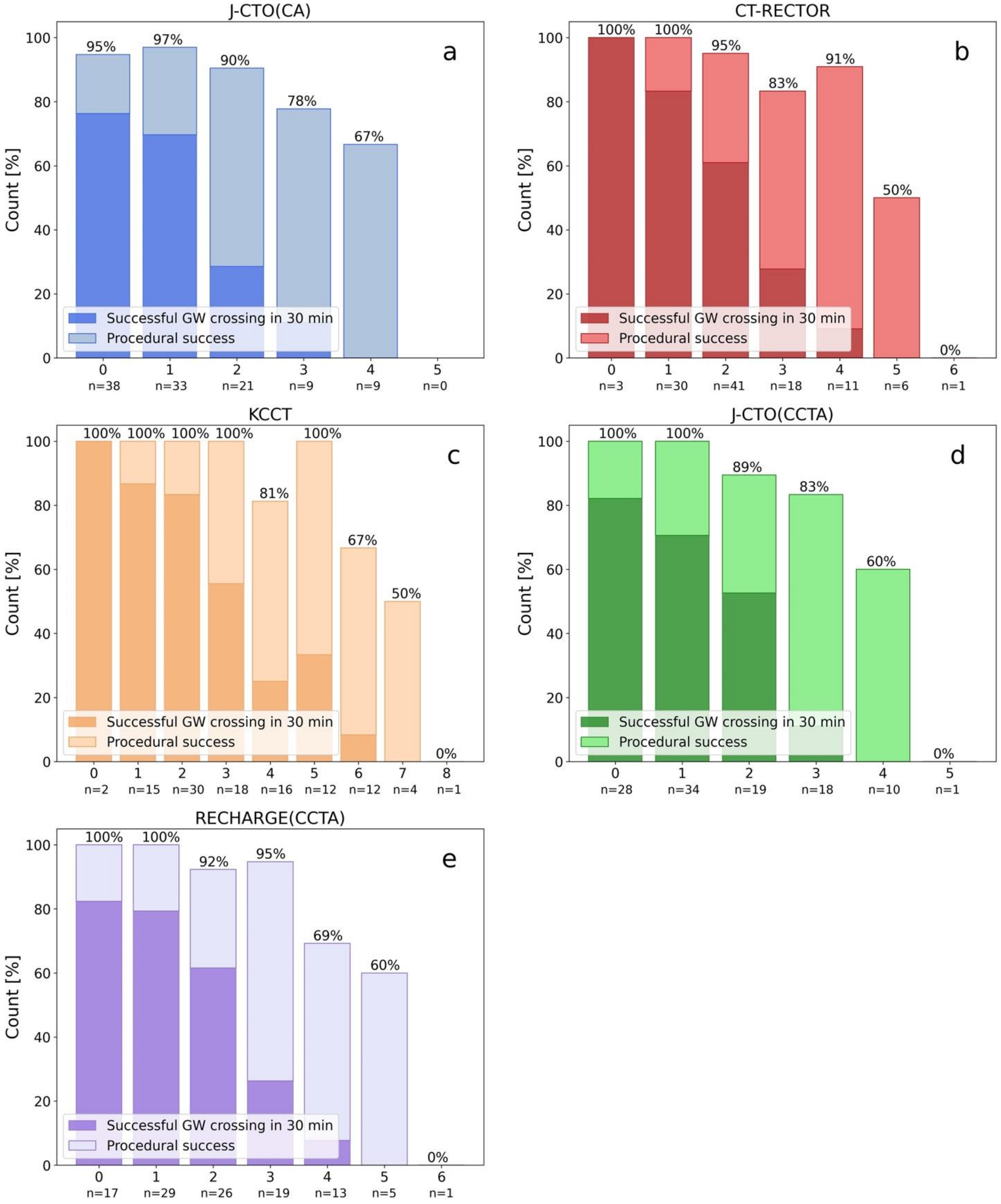

Figure S3. Time-efficient guidewire crossing and final procedural success rates depending on difficulty category based on (**a**) J-CTO$_{CA}$, (**b**) CT-RECTOR, (**c**) KCCT, (**d**) J-CTO$_{CCTA}$, and (**e**) RECHARGE$_{CCTA}$ scoring systems.

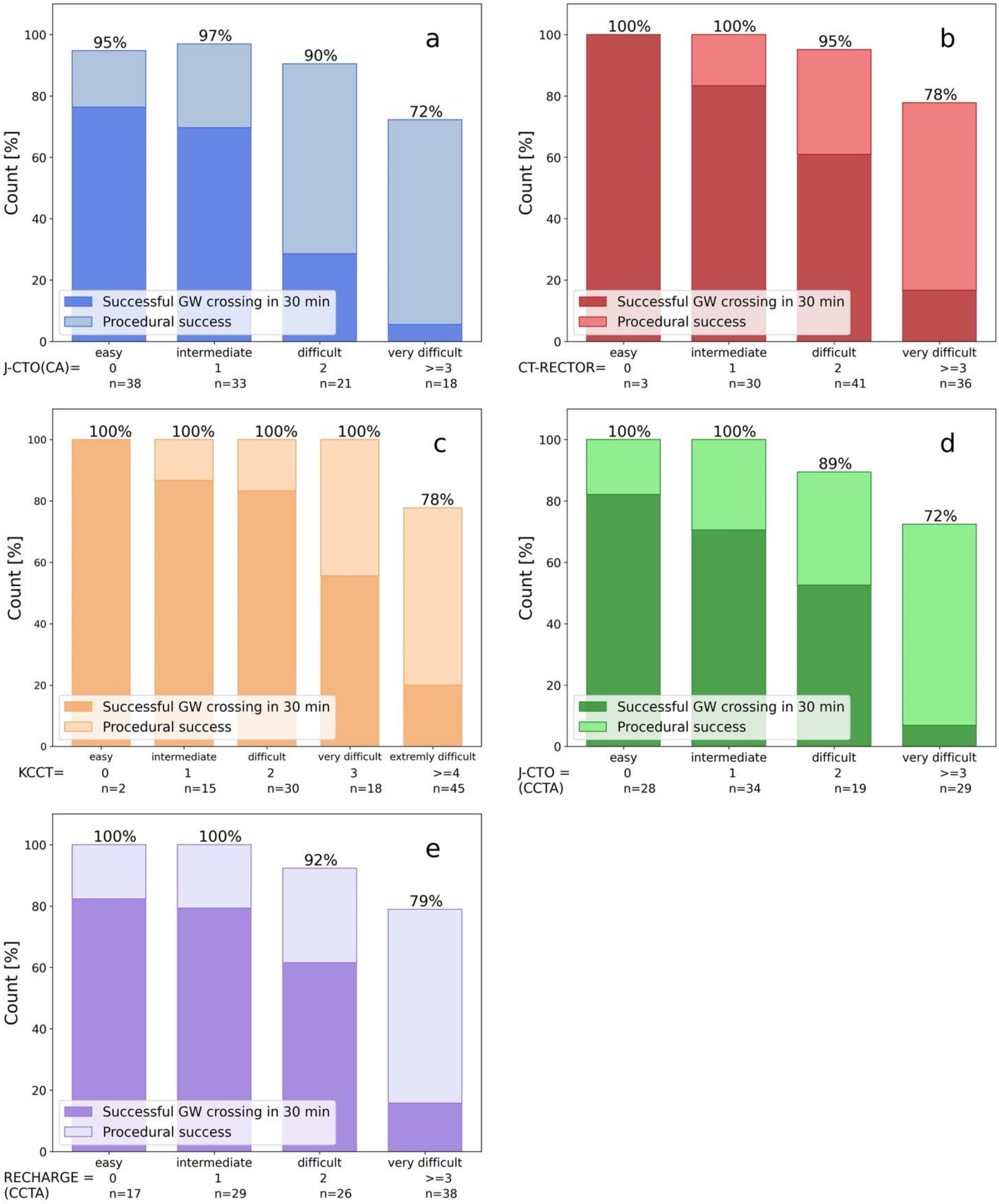

Supplement: Supplementary file 1 [file jcdd-11-00003-s001.zip › jcdd-2776753-supplementary.pdf]
